# Epigenetic reprogramming in cancer: From diagnosis to treatment

**DOI:** 10.3389/fcell.2023.1116805

**Published:** 2023-02-14

**Authors:** Pedro Mikael da Silva Costa, Sarah Leyenne Alves Sales, Daniel Pascoalino Pinheiro, Larissa Queiroz Pontes, Sarah Sant’Anna Maranhão, Claudia do Ó. Pessoa, Gilvan Pessoa Furtado, Cristiana Libardi Miranda Furtado

**Affiliations:** ^1^ Department of Physiology and Pharmacology, Drug Research and Development Center, Federal University of Ceará, Fortaleza, Ceará, Brazil; ^2^ Postgraduation Program in Biotechnology Northeastern Network of Biotechnology, Federal University of Ceará, Fortaleza, Ceará, Brazil; ^3^ Postgraduation Program in Pharmacology, Federal University of Ceará, Fortaleza, Ceará, Brazil; ^4^ Oswaldo Cruz Foundation, FIOCRUZ-Ceará, Sector of Biotechnology, Eusebio, Ceará, Brazil; ^5^ Postgraduation Program in Biotechnology and Natural Resources, Federal University of Ceará, Fortaleza, Ceará, Brazil; ^6^ Drug Research and Development Center, Postgraduate Program in Translational Medicine, Federal University of Ceará, Fortaleza, Ceará, Brazil; ^7^ Experimental Biology Center, University of Fortaleza, Fortaleza, Ceará, Brazil

**Keywords:** epigenetic reprograming, cancer, DNA methylation, histone modifications, non-coding RNAs, epidrugs

## Abstract

Disruption of the epigenetic program of gene expression is a hallmark of cancer that initiates and propagates tumorigenesis. Altered DNA methylation, histone modifications and ncRNAs expression are a feature of cancer cells. The dynamic epigenetic changes during oncogenic transformation are related to tumor heterogeneity, unlimited self-renewal and multi-lineage differentiation. This stem cell-like state or the aberrant reprogramming of cancer stem cells is the major challenge in treatment and drug resistance. Given the reversible nature of epigenetic modifications, the ability to restore the cancer epigenome through the inhibition of the epigenetic modifiers is a promising therapy for cancer treatment, either as a monotherapy or in combination with other anticancer therapies, including immunotherapies. Herein, we highlighted the main epigenetic alterations, their potential as a biomarker for early diagnosis and the epigenetic therapies approved for cancer treatment.

## Introduction

### Epigenetic reprogramming and cancer development

Since first described by Conrad Waddington in 1942 (Waddington, 1959), the epigenetic landscape during the differentiation process, where a totipotent undifferentiated cell acquires specialized characteristics and functions, is still a challenge for modern biology (Hunter, 2017). During early development, the whole genome is reprogrammed through epigenetic modifications such as DNA methylation, histone modification and non-coding RNA interaction, that alter chromatin structure and DNA accessibility by establishing a differential gene expression program in a cell-specific manner, without changes on DNA sequence (Skinner, 2011; Olynik and Rastegar, 2012). Epigenetic reprogramming is essential for normal development, as well as the maintenance of cell type-specific epigenetic patterns during cell division. However, given the dynamic and reversible characteristics of the epigenetic modifications, epigenetic reprogramming is strongly affected by environmental factors which play an essential role in the establishment and maintenance of epigenetic markers (Waddington, 1959).

Aberrant epigenetic reprogramming is associated with the etiology of developmental disorders as the imprinting defects (i.e., Beckwith–Wiedemann and Silver–Russell syndromes) (Monk et al., 2019), and complex and multifactorial diseases including metabolic syndrome (Ling and Rönn, 2019), cardiovascular diseases (Handy et al., 2011) and neurological disorders (Chheda and Gutmann, 2017). Due to its ability to regulate cell growth and differentiation pathways, non-mutational epigenetic reprogramming has been added as a hallmark of cancer (Hanahan, 2022) and maybe a driver mutational event in sporadic cancers that promotes genomic instability, tumor initiation and malignant transformation (Feinberg et al., 2006; Feinberg et al., 2016; Hanahan, 2022). These (epi)genetic changes confer a specific phenotype to cancer cells as the uncontrolled growth, resistance to death and increased invasiveness to adjacent tissues and/or spread to other organs (Ilango et al., 2020).

**FIGURE 1:**
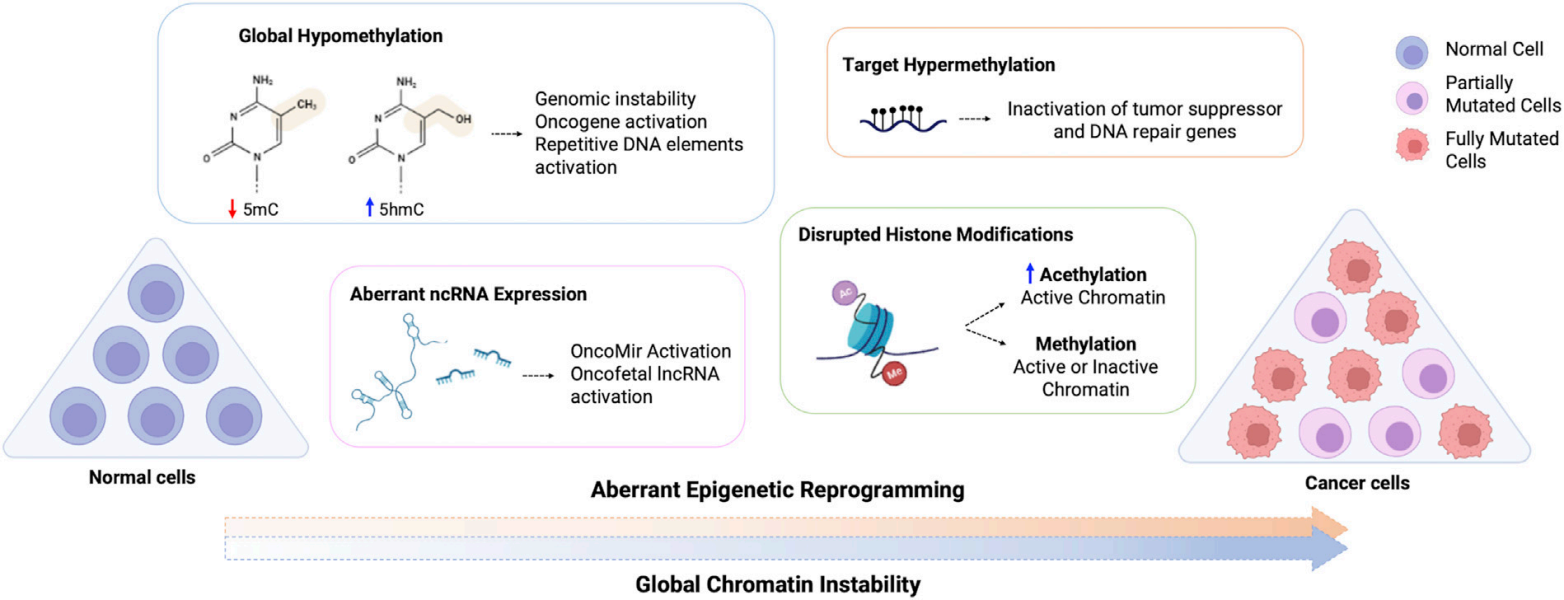
Schematic representation of the aberrant epigenetic reprogramming on cancer cells. Normal cells are aberrantly reprogramed through loss of global methylation, altered ncRNA expression, disrupted histone modifications and hypermethylation of target genes, which can inactivate tumor suppressor and DNA repair genes and activate oncogenes, resulting in the global chromatin instability observed in cancer cells. 5mC, 5-methylcytosine; 5hmC, 5-hydroxymethylcytosine.

Lifetime disruption in the epigenetic machinery leads to loss of global epigenetic marks, activation of growth-related genes (oncogenes) and silence of cell cycle control genes (tumor suppressor) and DNA repair genes, thereby initiating and propagating tumorigenesis (Figure 1) (Gibney and Nolan, 2010; Feinberg et al., 2016; Hanahan, 2022). These epigenetic features are similar to those observed in early development, where somatic cells are reprogrammed towards a less differentiated state followed by oncogenic reprogramming (Suvà et al., 2013; Feinberg, et al., 2016). The stem-like state or stem progenitor for cancer development is the major challenge in treatment, as it promotes unlimited self-renewal, multi-lineage differentiation and drug resistance (Suvà et al., 2013) Furthermore, alterations in the tumor microenvironment, tumor heterogeneity and regulation of stromal cells are associated with functional abilities acquired through epigenetic reprogramming (Ilango et al., 2020).

Because of the reversible nature of epigenetic changes, the possibility of reprogramming cancer epigenome has become a promising target for both treatment and reversibility of drug resistance (Miranda Furtado et al., 2019). The discovery of chemical compounds that act on the enzymes responsible for the maintenance and establishment of epigenetic mechanisms (epigenetic drugs), changing the epigenetic landscape of a tumor cell, has revolutionized cancer therapy, especially for hematological tumors (Montalvo-Casimiro et al., 2020; Morel et al., 2020). Moreover, the combination of epigenetic drugs with other anticancer therapies, such as chemo, hormonal or immunotherapy has broadened the perspective on the use of these compounds and their effectiveness on treatment. In this review, we present the epigenetic alterations in cancer reprogramming, the main epigenetic drugs and combining therapies for cancer treatment.

### Reprogramming DNA methylation in cancer

DNA methylation is an abundant epigenetic marker in the mammalian genome, that is stably maintained during DNA replication. Genome-wide loss of DNA methylation is a recognized epigenetic marker in oncogenic transformation, which is followed by an aberrant reprogramming of the cancer epigenome (Vandiver et al., 2015). The most common DNA modification is the chemical addition of a methyl group to the 5- carbon of the cytosine followed by a guanine (5′CG3′ or CpG, cytosine-phosphate-guanine), giving rise to 5-methylcytosine (5mC) (Geiman and Muegge, 2010). 5mC is a repressive epigenetic mark that aids in maintaining genomic stability as most CpG sequences in the genome are methylated (hypermethylated), except for CpG islands that are hypomethylated and usually encompass promoters and enhancers (Nishiyama and Nakanishi, 2021). This process is conserved from archaea to eukaryotes, from fertilization to every stage of development, and is carried out by the family of DNA methyltransferases (DNMTs). DNMT1, DNMT3A and DNMT3B are canonical methyltransferase enzymes, while DNMT2 and DNMT3L are non-canonical, without DNMT activity. DNMT1 is involved in the maintenance of DNA methylation, keeping the epigenetic memory in differentiated cells and restoring parental methylation patterns on the nascent DNA strand while DNMT3A and DNMT3B are related to *de novo* DNA methylation (Moore et al., 2013; Lyko, 2018). The role of DNMT2 and DNTM3L are not well understood, but they seem to act on RNA methylation activity and *de novo* DNA methylation, respectively (Schaefer et al., 2010; Veland et al., 2019). DNA methylation during semi-conservative DNA replication occurs with the support of the ubiquitin-like plant homeodomain and RING finger domain 1 (UHRF1) that recognizes CpGs in the hemimethylated DNA, and recruits DNMT1 to restore parental methylation patterns on the nascent strand (Hashimoto et al., 2008).

Global DNA hypermethylation is essential for the maintenance of genome stability, silencing of repetitive elements (transposon and retrotransposons) (Hur et al., 2014) and inactivation of nucleotide-repeat expansion (Poeta et al., 2020). Despite the high stability of DNA methylation, 5mC can be demethylated passively during DNA replication or actively through the oxidation of 5mC to 5-hydroxymethylcytosine (5hmC), with other intermediates (5-formylcytosine, 5fC and 5-carboxylcytosine, 5caC), by the ten-eleven translocated (TET) family of enzymes. The 5hmC is enriched at transcriptionally active regions, such as gene bodies and the borders of promoters and enhancers. The passive process is linked to the absence of DNMT1/UHRF1 which leads to the progressive dilution of cytosine methylation during successive rounds of DNA replication (An et al., 2017). Aberrant active demethylation and increased 5hmC marker at *TOP2A* and *EZH2* genes were associated with poor prognosis in an aggressive subtype of prostate cancer and are related to the activation of oncogenic pathways, such as *MYC* and *E2F*, and TGFβ signaling pathways in metastatic castration-resistant prostate cancer (Palanca-Ballester et al., 2021). On the other hand, loss of 5hmC marker seems to be a common alteration in penile (Rodriguez-Casanova et al., 2021) and oral squamous cell carcinoma (Wang et al., 2017) and myelodysplastic syndrome (MDS) (Cavalcante et al., 2022).

Regions of low CpG density are hypomethylated in cancer, leading to genomic instability thereby facilitating chromosomal rearrangements and DNA damage (Das and Singal, 2004; Baylin and Jones, 2016; Nishiyama and Nakanishi, 2021). However, once carcinogenesis is established, an increased 5mC marker is associated with tumor progression and reduced survival rate (Rodriguez-Casanova et al., 2021). Additionally, site-specific hypomethylation or hypermethylation promotes the activation of proto-oncogenes and the silencing of tumor suppressor genes, respectively (Baylin and Jones, 2011). Among these, genes related to cell cycle progression (e.g., *RB1*, *CDKN2A* and *CDKN2B*), invasion and metastasis (e.g., *CDH1* and *CDH13*) and apoptotic signaling (e.g., *DAPK1*) are hypermethylated in several cancers (Zafon et al., 2019; Nishiyama and Nakanishi, 2021). The transcription factor *MYC*, which is highly expressed during early development and silenced in somatic cells, is overexpressed in almost 70% of cancers (Slamon and Cline, 1984; Madden et al., 2021). Activation of this proto-oncogene by loss of DNA methylation is a common feature of tumor progression and aggressiveness (Souza et al., 2013). Increased chromatin accessibility at *MYC* locus is observed across different cancer types (Corces et al., 2018).

Biallelic expression of imprinted genes or loss of imprinting (LOI) is frequently observed in human cancers (Jelinic and Shaw, 2007), and is an early event for some tumors, such as the childhood Wilms’ tumor (WT) (Graf et al., 2021). The imprinting control regions (ICR) are differentially methylated (DMR) in a parent-of-origin manner, resulting in a monoallelic expression of the clustered genes. LOI at the H19/IGF2 locus, for example, is associated with WT in overgrowth syndromes (Graf et al., 2021), and other cancers such as bladder (Byun et al., 2007) and colorectal (Hidaka et al., 2018). LOI of KvDMR1, INPP5Fv2-DMR and RB1-DMR is also implicated in the pathogenesis of cancer (Rumbajan et al., 2013). A comprehensive analysis of The Cancer Genome Atlas showed that the imprinted genes *CDKN1C* and *PEG3* are downregulated in primary tumors, while the *MEST*, *PHLDA2* and *GNAS* were frequently upregulated. As the ICRs are enriched by long ncRNAs (lncRNAs), LOI promotes the overexpression of these regulatory elements during carcinogenesis (Kim et al., 2015).

Loss of function of DNMTs and TETs enzymes during cell differentiation and growth is also related to oncogenic transformation. Mutations in *DNMT3A*, *TET1/2* and *IDH1/2* are recurrent in leukemia and lymphoma (Guillamot et al., 2016). *TET2* mutations are associated with aberrant DNA methylation in a wide spectrum of myeloid malignancies, including myelodysplastic syndrome (MDS), myeloproliferative neoplasms (MPN), myelomonocytic leukemia (CMML), and acute myeloid leukemia (AML). Somatic mutations in *TET* family are linked to changes in the regulation of stem cell differentiation and transformation (Cimmino et al., 2011; An et al., 2017). *DNMT3A* mutations lead to loss of methylation and promote AML transformation, while abnormal CpG island hypermethylation dependent on DNMT3A is observed during AML progression (Spencer et al., 2017). *TET1* and *TET2* reduced expression levels have been frequently observed in hepatocellular carcinoma tissues (Wang P. et al., 2019). Mutational events at chromatin remodeling factors and *Wnt* signaling pathway promote aberrant DNA methylation pattern in human tumors (Saghafinia et al., 2018). Somatic mutations and microsatellite instability also affect cancer epigenome (Velho et al., 2014).

Besides the most common DNA methylation that occurs at CpG sites, non-CpG methylation at CpA and CpT are emerging epigenetic markers in eukaryotes. These modifications, especially the CpA methylation, which gave rise to N6-methyladenine (6mA), were first recognized in embryonic stem cells and seem to be mediated by the DNTM3A (Ramsahoye et al., 2000). Other enzymes may affect 6mA levels, such as the methyltransferase N6AMT1 and the demethylase ALKBH1 (Kweon et al., 2019; Shen et al., 2022). Although the low abundance of 6mA is observed in the mammalian genome under normal conditions, several studies indicated dynamic changes in 6mA levels during development and cancer. A lower abundance of the 6mA marker was observed in glioma cells (Kweon et al., 2019), whereas an increased 6mA was shown in hepatocellular carcinoma (Lin et al., 2022). A decrease of genomic DNA 6mA, accompanied by decreased methyltransferase N6AMT1 and increased demethylase ALKBH1 levels promotes tumorigenesis and is associated with poor prognosis in cancer patients (Xiao et al., 2018).

DNA methylation can also be detected in liquid biopsies, a minimally invasive procedure that has emerged as a promising element in cancer early detection. Liquid biopsies allow the monitoring of the molecular landscape of circulating tumor elements in body fluids in the search for new biomarkers of cancer diagnosis, prognosis and therapy (Martins et al., 2021; Michela, 2021). Cell-free circulating tumor DNA (ctDNA) exhibits both genetic and epigenetic cancer-related mutations making it possible to detect malignant lesions, monitor tumor evolution, metastasis and recurrence and predict treatment response (Luo et al., 2021; Li et al., 2022). Since aberrant DNA methylation is an early event in carcinogenesis, ctDNA methylation analysis has been used for cancer screening in clinical oncology. Considering that each cell group has a unique epigenetic signature, ctDNA methylation allows to tracing the origin of tissue in cancer patients (Luo et al., 2021).

Circulating SEPT9 methylation assay has been used as a CRC biomarker with increased specificity and sensitivity (DeVos et al., 2009; Church et al., 2014) and was the first blood-based screening approved by the FDA (Luo et al., 2021). *SHOX2* DNA methylation assay was used to distinguish between malignant and benign lung disease from bronchial aspirates (Schmidt et al., 2010). Afterwards, a plasma-based assay was used to detect small cell lung cancer and squamous cell carcinoma with high sensitivity (Kneip et al., 2011). Cell-free DNA (cfDNA) methylation of 15 DMRs allowed the detection and stratification between high and low-risk ovarian cancer (Liang et al., 2022). Similarly, other tumors, as breast, prostate and colorectal used cfDNA methylation to differentiate malignant and normal lesions, and prognosis stratification (Zhang et al., 2021c; Wu Q. et al., 2021; Chen et al., 2022a; Rodriguez-Casanova et al., 2022). Besides target ctDNA methylation, cell-free genome-wide 5hmC has been recently used for cancer diagnosis and prognosis stratification (Song et al., 2017; Xu et al., 2021; Shao et al., 2022). Despite the advances and promising use of cfDNA methylation in clinical oncology, there are some technical limitations that need to be addressed for use in clinical practice as a standard diagnostic tool (Heidrich et al., 2021; Lone et al., 2022).

DNA methylation has been widely investigated in oncology due to its control of time- and tissue-specific gene expression, inactivation of repetitive DNA and the maintenance of genomic stability during cancer initiation and progression. Further, the epigenetic reprogramming of undifferentiated cells through waves of DNA methylation and demethylation, can aberrantly reprogram the stem cell epigenome and promote cancer differentiation, recurrence, and resistance to treatment. Also, 5mC participates in the acquisition of other epigenetic markers as histone modification and ncRNA expression (Das and Singal, 2004; Geiman and Muegge, 2010). Herewith, DNA methylation is an important epigenetic marker for cancer diagnosis, with direct implications for survival rate and an emergent target for drug development.

### Aberrant histone modification

Another important epigenetic mark is the chemical modification of histone proteins. Histones assist DNA packaging into a highly organized chromatin structure. DNA is wrapped around a histone octamer (H2A, H2B, H3 and H4), linked by the histone H1 to form the nucleosome, a core structure of the chromatin (Lawrence et al., 2016). The amino-terminal tails of histone proteins are frequently subject to multivalent post-translational modifications (PTM), such as acetylation, phosphorylation, methylation and ubiquitination, altering the degree of local chromatin condensation, and consequently interfering in gene expression and DNA accessibility (Demetriadou et al., 2020). Non-conventional modifications can also occur in histones, such as citrullination/deamination, sumoylation, formylation and propionylation, among others (Tweedie-Cullen et al., 2012).

The most studied histone modifications are methylation and acetylation, however, unlike DNA methylation, histone modifications can either be an active or repressive epigenetic marker, depending on the modification and the modified amino acid group. Many enzymes catalyze methylation at histone protein, such as the histone methyltransferases (HMTs) and demethylase (HDM), lysine methyltransferases (KMTs) and demethylases (KDMs, also known as LSD), protein arginine methyltransferases (PRMTs), among others (Husmann and Gozani, 2019; Wu X. et al., 2021). This modification is frequently observed in lysine (K) and arginine (R) residues and is related to different transcriptional states (active or inactive) (Greer and Shi, 2012). Histone acetylation is a common modification in the lysine amino acid that is catalyzed by histone acetyltransferases (HATs) and histone deacetylases (HDACs). Acetylation, on the other hand, is usually an epigenetic marker of transcriptional activation (Bannister and Kouzarides, 2011).

The combination of these modifications is responsible for maintaining chromatin structure, which is dynamic and plays key role in development and cell differentiation. Aberrant reprogramming of histone modifications is often observed in the pathogenesis of cancer, changing chromatin accessibility, and altering the expression of target genes, subsequently affecting malignant progression (Zhao and Shilatifard, 2019). The imbalance of genome-wide histone methylation changes cell growth and may favor tumorigeneses. The enrichment of the tri-methylation at H3 lysine 9 (H3K9me3) and lysine 27 (H3K27me3), a repressive epigenetic marker found many promoters region, drives oncogenic transformation and chemoresistance (Torrano et al., 2019). Overexpression of *EZH2* (Enhancer of zeste homolog 2), a member of the polycomb proteins which is responsible for the tri-methylation of H3k27 and inactive chromatin state, is a marker of cancer initiation, progression, metastasis and targeted therapy (Chase and Cross, 2011; Duan et al., 2020). Although histone arginine methylation is a less typical and complex marker, increased expression of PRMT and enrichment of methylation at H3 arginine residues (H3R8, H3R3 and H3R2) is observed in digestive cancer cells (Chen et al., 2020).

Monoacetylation at H4K16 (H4K16ac) is a conserved marker that increases chromatin accessibility and gene activation. Reduced levels of the active H4K16ac and H4K20me3 histone modifications are a hallmark of cancer, perhaps by promoting the loss of DNA methylation at repetitive sequences (Fraga et al., 2005). The H3K27ac is often observed in active promoter and enhancer regions and is dysregulated with prognostic value for thyroid tumors (Zhang et al., 2021a). Increased active H3K27ac and reduced repressive H3K27me3 markers seem to be a driver mutation in high-grade gliomas, which is related to the activation of endogenous retroviruses (EVRs) and promotes therapeutic sensibility to demethylating agents (Krug et al., 2019). Histone H3 and H4 acetylation (H3K9, H3K18 and H4K12) and di-methylation (H4R3 and H3K4), an epigenetic marker of transcriptional activation, predicts molecular heterogeneity in prostate cancer with prognostic value (Seligson et al., 2005).

Post-translational modifications of histones, histone variants and histone-associated DNA modifications can be detected in liquid biopsies using circulating nucleosome (Bauden et al., 2015). Intact nucleosomes are released form cells after death and has been used as a biomarker for early diagnosis and monitoring of various types of tumors (Deligezer et al., 2010; Bauden et al., 2015; Van den Ackerveken et al., 2021). H4K20me3 and H3K27me3 histone modification at circulating nucleosomes is reduced in the plasma of patients with colorectal cancer (Gezer et al., 2015). H3K9me3 marker in the blood plasma was found decreased of patients with colorectal cancer and increased in patients with multiple myeloma (Deligezer et al., 2010). Elevated number of circulating nucleossome have been reported in many tumors (Bauden et al., 2015), and is associated with tumor recurrence and metastasis in breast cancer (Mego et al., 2020b; Mego et al., 2020a). Global analysis of histone PTMs showed 13 modifications specifically related o colorectal cancer and an increasing in methylation of histone H3K9 and H3K27, acetylation of histone H3 and citrullination of histone H2A1R3 (Van den Ackerveken et al., 2021).

The dynamic of histone modifications and modifiers changes chromatin landscape and genomic function and control cancer cell phenotype and promotes disease progression. Although the increased identification of histone modifications and the complex regulatory machinery made possible by the advancement of high-throughput technologies, much still needs to be revealed about the function of each modification and the implications for cancer development. Even though these modifications are predictive of clinical outcomes, targeting histone modifiers is a promising epigenetic therapy in anticancer drug discovery.

### Non-coding RNAs as cancer biomarkers

In the human genome, the protein-coding genes represent less than 2%, whereas a large fraction is constituted by regions that are transcribed into non-coding RNAs (ncRNAs), which retain fundamental biological properties within cells, controlling gene expression in a cell-specific manner. Besides its function in the regulation of transcription, ncRNAs also influence the translation as components in the protein synthesis machinery and regulate other ncRNAs function in a complex network (Ratti et al., 2020). Therefore, roles of ncRNAs in physiology and pathology are recognized, including developmental, gametogenesis, stress, immune response, and tumorigenesis (Aprile et al., 2020; Ratti et al., 2020; Taniue and Akimitsu, 2021).

Among ncRNAs, the small ncRNAs (<200 nucleotides, nt) and long ncRNAs (>200 nt) have important roles in cancer development, acting both as oncogenic and tumor suppressor molecules (Ratti et al., 2020). MicroRNAs (miRNA) and small interfering RNA (siRNAs), are small ncRNA duplex, approximately 18–31 nucleotides long, that regulates gene expression at the post-transcriptional level through target block of translation and/or degradation (Zhang et al., 2021b; Torsin et al., 2021). Both miRNA and siRNA have been extensively investigated as molecular markers of cancer and therapeutic agents. The main difference is that the siRNA is highly specific to the target, while the miRNA can target several molecules simultaneously, regulating multiple pathways to maintain physiological homeostasis (Lam et al., 2015; Cuciniello et al., 2021). Aberrant miRNA expression has been reported in several cancer types, inducing cell proliferation, invasion, and resistance to death by activating oncogenes and silencing tumor suppressor genes (Peng and Croce, 2016).

Long non-coding RNAs (lncRNAs, >200 nt) are the most abundant class of ncRNAs in the human genome (Slack and Chinnaiyan, 2019). LncRNAs arise from intergenic regions or are clustered with protein-coding genes (intronic or in gene-dense regions) and like protein-coding genes, their promoter regions are globally enriched with histone modifications, such as H3K27ac, H3K4me3 and H3K9ac (Quinn and Chang, 2016; Taniue and Akimitsu, 2021). The abundant class of lncRNAs modulate gene expression in a complex intracellular network of crossed interactions (competing endogenous RNA networks, or ceRNET) through chromatin remodeling, either as *cis* or *trans* elements, targeting specific sequences at the transcriptional and translational level and participating in post-translational modifications (Engreitz et al., 2016; Yao et al., 2019). Therefore, the lncRNAs, and their protein-and RNA-based regulation, added complexity to the cytoplasmatic post-transcriptional and translation control, orchestrated before by miRNAs and proteins (Aprile et al., 2020).

Many lncRNAs are highly expressed during development and participate in cell growth and differentiation pathways (Cabili et al., 2011). Thus, their modulation in different intracellular pathways, such as cell survival and proliferation, glucose metabolism, apoptosis, metastasis formation, and drug resistance, results in the tumor phenotype (Luo et al., 2018; Hu et al., 2020; Wan et al., 2020; Liang et al., 2022). Disruption of lnRNA expression or stability affects the expression of the neighboring genes (Horlbeck et al., 2020) and promotes chromosomal rearrangements (Yin et al., 2021) modulating several hallmarks of cancer and fostering progression. Moreover, several lncRNAs are transcriptionally regulated by oncoproteins or tumor suppressors, which are directly related to tumorigenesis (Guttman et al., 2009; Taniue et al., 2016).

Other classes of ncRNAs, such as small nucleolar RNA (snoRNAs), involved in RNA modifications and ribosome biogenesis; small nuclear RNAs (snRNAs), involved in pre-mRNA processing; piwi-interacting RNAs (pi-RNAs), which is mainly functional in the germline, inhibiting the transcription and movement of retrotransposons, repetitive sequences, and other mobile elements, have also been implied in cancer development and progression (Zhang et al., 2021b; Xiao et al., 2022). Moreover, circular RNAs (circRNAs), single-stranded covalently closed RNA loops, which act as transcriptional regulators, miRNA sponges and splicing and protein translation regulators, are also abundant in cell cytoplasm, and widely distributed in body fluids and cell-free samples, playing critical roles in tumorigenesis (Zhao et al., 2021).

Besides the main function of ncRNAs in cellular compartments, ncRNAs can also be released from the cell and transported in body fluids through exosomes or RNA binding proteins (RBPs), targeting molecules outside the production site, being a promising minimally invasive cancer biomarker (Qi et al., 2016). Circulating RNAs (as ncRNAs) are easy to be sampled in liquid biopsies and can be used as diagnostic biomarkers in the early detection of cancer, before radiologic and imaging events, as well as for prognosis, monitoring disease evolution and adjustments of treatment (Pardini et al., 2019). Due to their specificity and stability, circulating ncRNAs can provide accuracy and sensitivity for the screening of different human cancers (Slack and Chinnaiyan, 2019). Indeed, there are a large variety of ncRNAs that might be used as cancer biomarkers in liquid biopsies. Among them, the most studied are miRNAs, but more recently also pi-RNAs, circRNAs, and other sncRNAs. The lncRNAs also represent a versatile and promising group of molecules which, besides their use as biomarkers, have also a possible therapeutic role (Pardini et al., 2019).

One of the first described miRNA, and probably the most recurrently detected as a cancer biomarker, is the oncomiR miR-21. This miRNA is implicated in various signaling pathways, and its upregulation results in the inactivation of several tumor suppressors. Altered miR-21 expression is often observed in many cancer types, including digestive, respiratory, hematological, gynecological and brain malignancies (Kumarswamy et al., 2011; Pardini et al., 2019). MiR-21 was also upregulated in serum samples from patients with renal cell carcinoma (RCC), as well as miR-210 and miR-144-3p, and the upregulation of miR-21 was positively correlated to tumor stage (Zhao et al., 2013; Lou et al., 2017; Tusong et al., 2017). On the contrary, miR-508-3p and miR-509-5p were decreased in plasma samples of RCC patients (Zhai et al., 2012). Furthermore, miR-155 has been also reported as dysregulated in serum/plasma samples in gastroenterology malignancies, lung, breast, ovary, and hematologic malignancies (Larrea et al., 2016; Chen et al., 2017; Giannopoulou et al., 2019). Likewise, miR-141 and miR-375 were upregulated in serum and plasma exosomes of patients with metastatic prostate cancer (Hessvik et al., 2013; Samsonov et al., 2016). Although promising, miRNAs are not specific to one type of cancer, therefore understanding the implication of miRNAs in specific pathways and finding the most sensitive and specific ones is still challenging (Pardini et al., 2019).

LncRNAs, particularly circular lncRNAs, are stable circulating ncRNAs used as cancer biomarkers. In this context, overexpression of the oncogenic lncRNA MALAT1 in non-small cell lung cancer (NSCLC) tissue is related to reduced overall survival and could be a potential prognostic biomarker and therapeutic target in early-stage lung cancer (LC) (Gutschner et al., 2013; Huang et al., 2017; Lu et al., 2018). Otherwise, circulating MALAT1 expression is lower in patients with LC when compared to healthy controls (Weber et al., 2013; Guo et al., 2015). Another lncRNA overexpressed in LC is the imprinted gene *H19* which is associated with carcinogenesis from early stages to metastasis, reduced disease-free survival (DFS) time, and poor prognosis. Plasma level of the lncRNA H19 is also increased in NSCLC patients (Ge and Yu, 2013; Luo et al., 2018; Yin et al., 2018). In the same context, the imprinted lncRNA KCNQ1OT1 (LIT1) is dysregulated in human tumors (Nakano et al., 2006), and seems to be related to chemoresistance in tongue squamous cell carcinoma and poor prognosis (Guo et al., 2014).

Wang and collaborators demonstrated that the lncRNA colon cancer-associated transcript 2 (CCAT2) was significantly overexpressed in colorectal cancer (CRC) tissues of CRC patients when compared to healthy controls. Overexpression of CCAT2 was also seen in serum and serum-derived exosomes of the CRC patients (Wang L. et al., 2019). Likewise, in AML, the lncRNAs SBF2-AS1, DANCR, LINC00239, LINC00319, LINC00265 and LEF1-AS1 are overexpressed. Whereas the lncRNA H22954 is downregulated, and its decreased expression is related to a higher risk of relapse (Zimta et al., 2019). In gastric cancer (GC), different lncRNAs are related to drug resistance, by modulating the expression of drug resistance-related genes, such as the oncogenic lncRNAs MACC1-AS1, PVT1 and HAGLR, which are upregulated and promote 5-FU resistance in GC cells (Liu et al., 2022).

CircRNAs have been reported to play important roles in cancer growth, metastasis, and resistance to therapy (Su et al., 2019). In this context, Zhang et al. reported that circUBAP2 was overexpressed in osteosarcoma cells, and its knockdown inhibited cell proliferation and promoted cell apoptosis. CircUBAP2 acts inhibiting the expression of miR-143, thus enhancing the expression of Bcl-2, an important anti-apoptotic molecule (Zhang et al., 2017a). Likewise, circNFIX was found overexpressed in glioma and inhibited apoptosis through sponging miR-34a-5p, regulating NOTCH1 expression (Xu et al., 2018). In NSCLC, circSNAP47 expression, through the miR-1287/GAGE axis, is correlated with metastasis and associated with decreased overall survival (Li et al., 2018). On the other hand, circSHPRH in NSCLC is associated with downregulated metastasis and improved overall survival (Liu et al., 2018).

Due to the important role of ncRNAs in many biological processes through chromatin remodeling, gene expression regulation, protein synthesis and post-translational modifications, these regulatory RNAs have emerged as an important biomarker for cancer diagnosis, with implications in disease prognosis, drug resistance and targeted therapy. Additionally, the possibility to detect small and long ncRNAs in cell-free body fluids and liquid biopsies, circulating ncRNAs represents a new class of minimally invasive biomarkers for the early diagnosis of cancer.

### Epigenetic modifications in ncRNAs

RNA modifications have emerged as important post-transcriptional regulators of gene expression patterns and have shown significant implications in several human diseases, including cancer (Barbieri and Kouzarides, 2020; Nombela et al., 2021). These modifications can be divided into two categories: reversible, which include chemical modifications, i.e., the different types of RNA methylation, such as cytosine and adenosine methylation; and non-reversible, i.e., editing and splicing, including the formation of circular RNAs (Esteller and Pandolfi, 2017). Most of the time, those chemical modifications are dynamic, as a result of adaptation to the cell environment, however, they can also be transmitted during mitosis and meiosis.

The epitranscriptome scenery is complex, and more than 170 different types of chemical modifications are described for coding and ncRNAs (Boccaletto et al., 2018). A familiar chemical modification of some RNAs affects its 5′-end, the well-known “5′cap”, and the most characterized cap modification is the addition of an N^7^-methylguanosine (m^7^G) (Ramanathan et al., 2016). Another frequent modification in RNAs is the 5-methylcytosine (m^5^C), first thought to be present only in tRNAs and rRNAs, and later identified in other RNA transcripts, which might have a role in miRNA targeting (Squires et al., 2012). The lncRNA XIST, for example, is regulated by m^5^C where cytosine methylation has been shown to interfere with the binding of the histone modifier PRC2 (Amort et al., 2013). Importantly, m^5^C is not a static mark of RNA, and can be demethylated to 5-hydroxymethylcytosine. RNA can also be modified at adenosines in the form of N^6^-methyladenosine (m^6^A) and N^1^-methyladenosine (m^1^A). m^6^A is the most abundant internal modification of mRNA (Lee et al., 2014), but it is also relevant for miRNAs controlling their maturation and expression levels. m^6^A is also found in lncRNAs, being required, for example, for the efficient transcriptional repression mediated by the lncRNA XIST and modulating the structure of lncRNA MALAT1, which is associated with cancer malignancy (Patil et al., 2016; Zhang et al., 2017b). Pseudouridylation, the 5′-ribosyluracil isomers of uridine, is a common modification in ncRNAs, including tRNA, rRNA and snoRNAs and lncRNAs (Ge and Yu, 2013; Schwartz et al., 2014).

All those RNA modifications are dynamic, allowing rapid cellular responses to environmental signals, and finely regulating several molecular processes within the cell: altering RNA metabolism, splicing or translation; RNA stability or intracellular localization; binding affinity to RBPs or other RNAs; and finally diversifying (epi)-genetic information (García-Vílchez et al., 2019; Gkatza et al., 2019; Nombela et al., 2021). Although RNA modifications are not alone considered cancer drivers, the resulting ability of them to modulate several processes of RNA metabolism, leads to aberrant expression of important genes functionally related to survival proliferation, self-renewal, differentiation, migration, stress adaptation, and resistance to therapy, all of which are hallmarks of cancer. Alterations in the expression of m^6^A writers (i.e. METTL3 and METTL14), erasers (i.e. FTO) or readers (i.e. YTHDC2 and YTHDF1), for example, are associated with tumor-suppressive or tumor-promoting scenarios (Blanco et al., 2016; Cui et al., 2017; Jin et al., 2019; Nombela et al., 2021).

In hepatocellular carcinoma (HCC), for example, the mutation frequency of m^5^C regulatory genes is high, and their dysregulation is associated with higher stages of HCC (He et al., 2020). In bladder cancer, NSUN2 and m^5^C reader YBX1 are upregulated, which are positively correlated with T and N stages, and poor disease-free survival in those patients (Chen et al., 2019). In breast cancer cell lines, 2′-O methylation appeared to be hypermodified in rRNA and correlated with altered protein translation (Belin et al., 2009). Modifications in tRNA, which includes m^5^C or 5-methoxycarbonylmethyluridine (mcm^5^U), have been also reported in breast cancer, and correlate with altered translation (Begley et al., 2013; Delaunay et al., 2016; Nombela et al., 2021). In bladder cancer cells, METTL3 promotes the maturation of miR-221/222 in an m^6^A-dependent manner, which causes PTEN reduction, leading to cell proliferation and tumor growth (Han et al., 2019). In HCC, METTL14 promotes the m^6^A-dependent processing of pri-miR-126, and its depletion reduces m^6^A levels and expression of miR-126, leading to cancer cell migration and invasion (Ma et al., 2017). In nasopharyngeal carcinoma (NPC), the oncogenic lncRNA FAM225A stabilized by m^6^A modifications serve as a sponge for miR-590-3p and miR-1275, activating the FAK/PI3K/Akt signaling pathway and promoting tumorigenesis and metastasis (Zheng et al., 2019). Therefore, strategies aiming these aberrant post-transcriptional RNA modifications in cancer cells may be an efficient targeted therapy for tumors.

### Epigenetic treatment in cancer

**FIGURE 2:**
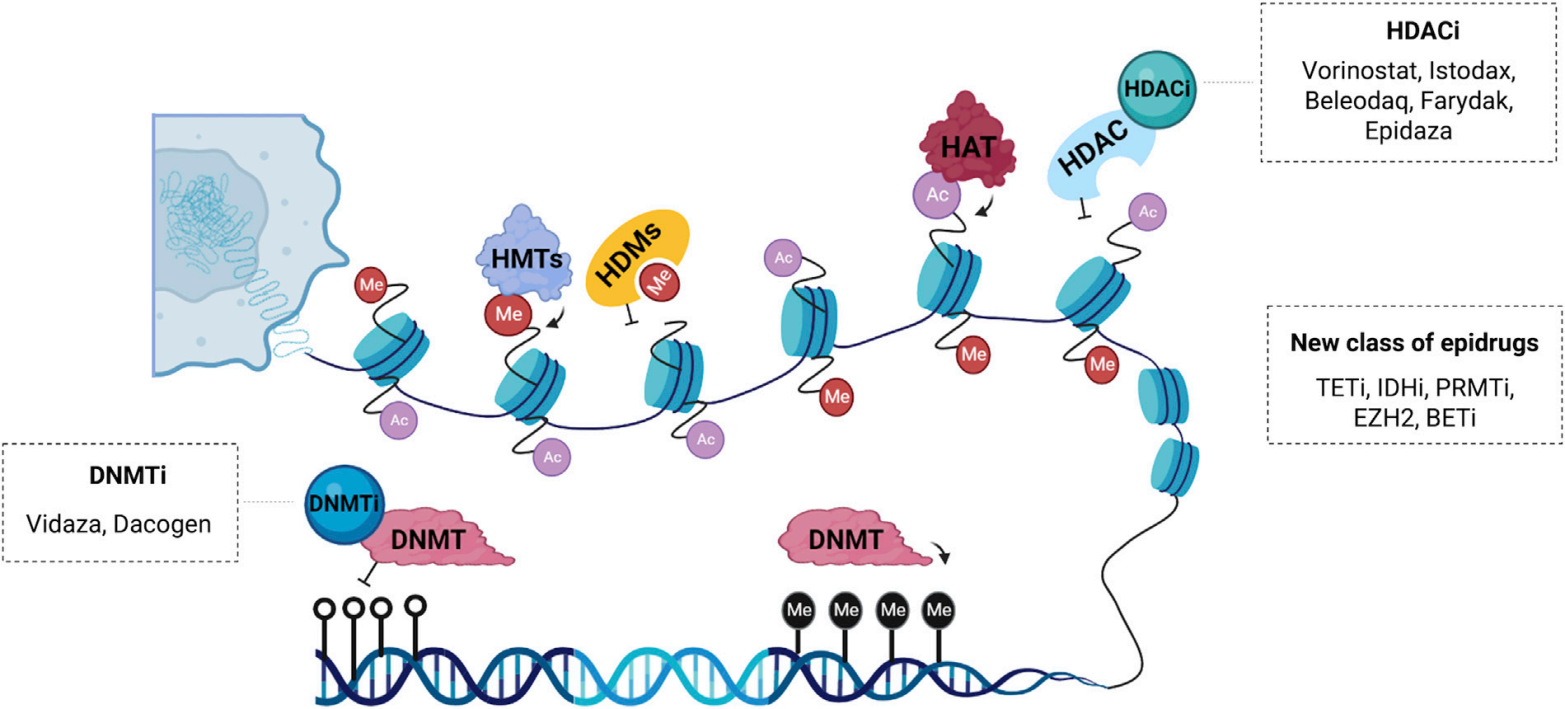
Schematic representation of the main epidrugs targets. Histone acetyltransferases (HATs) and deacetylases (HDACs) are enzymes responsible for post-translational acetylation and deacetylation, respectively. HDAC inhibitors (HDACi) such as Vorinostat^®^, Istodax^®^, Beleodaq^®^, Farydak^®^ and Epidaza^®^ induce acetylation thereby promoting transcriptional activation. Histone methyltransferases (HMTs) and demethylases (HDMs) can also be modulated by a new class of epigenetic drugs. DNA methyltransferases (DNMTs) are enzymes responsible for transferring a methyl group to carbon five of cytosine, a repressive epigenetic marker. DNMT inhibitors (DNMTi) like Vidaza^®^ and Dacogen^®^ promote loss of methylation and activation of aberrantly silenced genes. Other compounds with epigenetic activity are inhibitors of the enzyme ten-eleven translocation (TETi), inhibitors of the enzyme isocitrate dehydrogenase (IDHi), inhibitors of the protein arginine methyltransferase (PRMTi), inhibitors of the bromodomain and extra-terminal domain (BETi) and inhibitors of the enhancer of zeste homolog 2 (EZH2i), which is a histone-lysine n-methyltransferase enzyme.

Genetic changes, including genetic mutations, are difficult to reverse, unlike epigenetic modifications which are reversible and can be modulated by pharmacological agents (Zhang et al., 2020). The epigenomic´s reprogramming, leading to changes in the cell landscape, reveals a promising therapeutical approach (Miranda Furtado et al., 2019). Many small molecules targeting epigenetic key enzymes, called epigenetic drugs or epidrugs (Table 1), have been discovered and new compounds that modulate epigenetic marks (Figure 2) are being developed focusing on cancer treatment (Xiao et al., 2021) (Jin et al., 2022b). Epidrugs promotes disruption of transcriptional and post-transcriptional modifications, acting manly on tumor suppressor and DNA repair gene activation (Rodríguez-Paredes and Esteller, 2011; Ghasemi, 2020). These drugs are tumor and disease stage specific and the side effects are mostly related to hematologic disorders like leukopenia, neutropenia and thrombocytopenia, and gastrointestinal symptoms as nausea, emesis, diarrhea and constipation (Table 1) (San Miguel Amigo et al., 2011; Bubna, 2015). Most of the side effects are reversed after treatment cessation and taper off with the use of appropriate medicines (Götze et al., 2010; Dong et al., 2012).

**TABLE 1:** Epigenetic drugs approved by FDA.

Epigenetic target	Compound	Clinical name	Disease	Administration	Side effects	Company	Approved by (Year)
DNMT1	Azacitidine	Vidaza^®^	MDS	Intravenous	Neutropenia, thrombocytopenia, nausea, emesis, diarrhea, and constipation	Pharmion Corporation	United States FDA (2004)
	Decitabine	Dacogen^®^	MDS	Intravenous	Prolonged myelosuppression (neutropenia and thrombocytopenia)	Janssen Pharmaceuticals	United States FDA (2006)
HDACs class I and HDAC6	Suberoylanilide hydroxamic acid (SAHA)	Vorinostat^®^	CTCL	Oral	Fatigue, nausea, diarrhea, and thrombocytopenia	Merck	United States FDA (2006)
HDAC6	Romidepsin	Istodax^®^	CTCL	Intravenous	Fatigue, nausea, leukopenia, granulocytopenia, and thrombocytopenia	Celgene	United States FDA (2009)
pan-HDACi	Belinostat	Beleodaq^®^	T-cell lymphoma	Intravenous	Nausea, vomiting, fatigue, pyrexia, and anemia	Topo Target	United States. FDA (2014)
	Panobinostat	Farydak^®^	Multiple myeloma	Oral	Diarrhea, peripheral neuropathy, asthenia, fatigue, neutropenia, thrombocytopenia, and lymphocytopenia	Novartis	United States FDA (2015)
HDAC1, HDAC2 HDAC3 and HDAC10	Tucidinostat	Epidaza^®^	PTCL	Oral	Fatigue, anorexia, thrombocytopenia, leukopenia, neutropenia	Chipscreen Biosciences	China FDA (2014)

DNMT, DNA, methyltransferase; HDAC, histone deacetylases; MDS, myelodysplastic syndrome; CTCL, Cutaneous T-cell lymphoma PTCL, Peripheral T-cell lymphoma; U.S, united states; FDA, Food and Drug Administration.

The first epigenetic drug approved by the United States Food and Drug Administration (FDA) was azacitidine (Vidaza^®^) in 2004 for MDS and chronic myelomonocytic leukemia (Kaminskas et al., 2005), followed by decitabine (Dacogen^®^) approved in 2006 to treat MDS (Steensma, 2009). Both azacitidine and decitabine are two analogues of the cytidine nucleoside in which the carbon atom in position 5, in the pyrimidine ring, has been replaced by a nitrogen (Derissen et al., 2013). Initially, these compounds were planned as cytotoxic agents, but it was found that low dose exposition could cause DNA demethylation by inhibiting the DNMT1 enzyme responsible for maintaining DNA methylation (Stresemann and Lyko, 2008). These so-called ‘hypomethylating agents’ have been used in myeloid malignancies for more than 1 decade, even though, 50% of patients do not respond initially or during repeated cycles of treatment (Zhao et al., 2021; Šimoničová et al., 2022).

Guadecitabine is a second-generation DNA methylation inhibitor being developed for AML and MDS treatment. It consists of a dinucleotide of decitabine and deoxyguanosine which is resistant to cytidine deaminase, the enzyme which is responsible for decitabine inactivation (Stomper et al., 2021). Guadecitabine might replace azacitidine and decitabine in a near future, due to its higher stability, safety profile and ease of administration (subcutaneous) (Daher-Reyes et al., 2019). Another class of epigenetic drugs are the histone deacetylase inhibitors (HDACi) which increase histone acetylation, an epigenetic mark of transcriptional activation, leading to an accessible chromatin conformation and promoting the expression of important genes that controls cell growth and death (Richon et al., 2009; Ramaiah et al., 2021).

The first HDACi was suberoylanilide hydroxamic acid (SAHA, vorinostat^®^) approved in 2006 by the FDA for the treatment of cutaneous manifestations of T-cell lymphoma (CTCL) (Duvic and Vu, 2007). After SAHA approval, a depsipeptide natural product from the bacterium *Chromobacterium violaceum* named romidepsin was approved in 2006 for CTCL and peripheral T-cell lymphomas (PTCL) treatment (McClure et al., 2018). Belinostat became the third FDA approved HDACi for T-cell lymphoma (Campbell and Thomas, 2017). In 2015, panobinostat arise as the first HDACi approved for a nonlymphoma cancer by FDA and also the European Medicines Agency (EMA). Panobinostat is an oral pan-HDACi recommended for relapsed or refractory multiple myeloma management (Berdeja et al., 2021), the first time that an HDACi was proposed and accepted for non-lymphoma cancer treatment, refreshing the possibility of designing inhibitors for all HDACs bearable enough to benefit humans (McClure et al., 2018).

Tucidinostat (chidamide) is a novel oral subtype of selective HDACi. This drug inhibits class I HDACs (HDAC1, HDAC2, HDAC3) and class IIb (HDAC10). It was approved in 2014 as a second-line therapy for peripheral relapsed or refractory T-cell lymphoma by the China Food and Drug Administration. In Japan, tucidinostat was approved in 2021 for relapsed or refractory adult T-cell lymphoma treatment under the name Hiyasta (Sun et al., 2022). Valproic acid is an FDA-approved antiepileptic drug that also presents inhibitory HDAC class I and II activity (Lunke et al., 2021). Currently, valproic acid is in phase III clinical trial as a potential drug to treat cervical and ovarian malignancies and has been proposed in combination regimens with chemotherapy and radiotherapy (Krauze et al., 2015; Suraweera et al., 2018; Tsai et al., 2021).

Compounds that modulate epigenome are being discovered and currently, there is a race in finding potential inhibitors of epigenetic modifiers. Emerging targets that modulate others DNA-modifying enzymes, as TETs and isocitrate dehydrogenase (IDHs) inhibitors (TETi and IDHi) are in current development for cancer treatment. Likewise, the complex network of histone-modifying enzymes has been added in anticancer therapy, as HMTi, HATi*,* HDMi, KMTi and PRMTi (Ganesan et al., 2019; Morel et al., 2020). An emerging target therapy for cancer treatment in preclinical studies is the EZH2 lysine methyltransferase inhibitors, with great results especially in combination with radiotherapy or chemotherapy, such as cisplatin, gefitinib and tamoxifen (Duan et al., 2020; Morel et al., 2020). Another class of epidrugs are the inhibitors of bromodomain and extra-terminal domain (BETi), a histone “reader” that recognizes and binds to acetylated lysine and is responsible for the recruitment of transcription machinery and gene activation (Cheung et al., 2021).

The combined use of epigenetic drugs with conventional therapies is gaining prominence due to its potential in increasing tumor cells' sensitivity to classical chemotherapy improving the therapeutical effect. A phase Ib/II clinical trial showed that the small molecule eprenetapopt in combination with azacytidine improved clinical response rates and molecular remissions in patients with *TP53* mutant MDS and oligoblastic AML (Sallman et al., 2021). A selective BCL-2 inhibitor (Venetoclax) has recently been approved for use in combination with hypomethylating agents (Azacitidine or Decitabine), giving promising results for the treatment of acute myeloid leukemia in patients who are ineligible to receive intensive chemotherapy (Manda et al., 2021). The use of decitabine associated with carboplatin has shown greater efficacy in the treatment of ovarian cancer when compared with conventional therapies by increasing the sensitivity of the tumor cells (Fan et al., 2014; Fu et al., 2015).

In the same context, a phase II study showed that patients with AML or MDS who underwent idarubicin therapy with a high-dose continuous infusion of Ara-C (cytarabine) associated with vorinostat^®^ showed an overall response rate of 85%, and a 76% complete response to treatment in association (Garcia-Manero et al., 2012). A multicenter phase 2 trial showed that the combined use of Vorinostat^®^ with Bortezomib and Dexamethasone showed an overall response of 81.3% in patients with relapsed multiple myeloma, although more studies are necessary to further optimize HDACi-based combinations, in order to improve tolerability and increase the efficacy of combination therapy (Brown et al., 2021). A multicenter phase II trial showed that the triple use of belinostat, carboplatin and paclitaxel was well tolerated and demonstrated clinical benefit in patients with recurrent epithelial ovarian cancer (Finkler et al., 2008). Combination therapy of drugs with the ability to modulate the epigenome and conventional therapies demonstrate increased efficacy and tolerability, requiring lower dosages of each agent, and reducing the side effects caused by conventional chemotherapies (Oing et al., 2019).

Recently, ncRNA have been proposed as a target to overcome therapy resistance. The focus of this approach is to inhibit the specific ncRNA molecule if it is overexpressed or restore the normal function of ncRNAs that are downregulated when therapy resistance occurs (Chen et al., 2022b). Inhibition of the microRNA-21 (miR-21) with a locked-nucleic acid-anti-miR resulted in increased apoptosis level in melanoma cell line and reduced tumor growth and volume in mice (Javanmard et al., 2020). Targeted inhibition of miR-221/222 with anti-miR-221 and anti-miR-222 promoted synergetic effects stimulating cell sensitivity to cisplatin in triple-negative breast cancer cell line (Li et al., 2020).

### Epidrug-associated immunotherapy

Immunotherapy refers to the treatment in which the patient’s immune system is reprogrammed and stimulated to fight defective cells such as those resulting from a tumorigenic process. Its main goal is to empower immunity and modulate the tumor microenvironment by releasing cytokines such as interferons, interleukins, and chemokines, promoting T-cell attack and tumoral cell cleaning (Esfahani et al., 2020).

Monoclonal antibodies have been an important therapeutic agent used in the treatment of several types of cancer. Since the approval in 1997 of Rituximab, the first therapeutic antibody approved for oncology patients and used until the present day with success in the treatment of B-cell malignancies, dozens of antibodies recognizing a variety of targets have been used successfully in the treatment of both solid and hematological tumors (Zahavi and Weiner, 2020). New antibody formats have emerged such as antibody-drug conjugates (ADCs), bispecific/multispecific binding, nanobodies, antibody fragments, and other engineered molecules (Jin et al., 2022a). In addition to monotherapy, antibodies can be combined with other drugs since multiple treatments can increase their effectiveness and decrease chemoresistance (Miranda Furtado et al., 2019).

Epidrugs are alternative strategies for a more personalized tumor treatment because they might sensitize tumors to immune checkpoint inhibitors and cell therapy, besides their effect on viral mimicry response and immune cell activation. Currently, several clinical trials on different tumor types are ongoing using epidrugs alone or in combination with other immunotherapy drugs (Xu et al., 2022). There are different approaches regarding the use of epidrugs in cancer treatment, such as DNA methyltransferase inhibitors (DNMTi), histone deacetylase inhibitors HDACi, and bromodomain and extra-terminal motif inhibitors BETi.

New combinations of epidrugs and immunotherapeutic molecules have shown potential clinical application for cancer treatment. A study of colorectal cancer in CT26 tumor-bearing mouse model revealed a therapeutic gain when low-dose decitabine was administrated with anti-PD-1 regimen (Yu et al., 2019). Decitabine is a well-known DNMTi and FDA-approved drug. A methylation profile was assessed in decitabine-treated CT26 cells and patient-derived xenografts (PDX) model, and tumor cells were significantly downregulated regarding methylation of promoter regions after decitabine treatment. It was also observed that decitabine in combination with anti-PD-1 antibody administration promoted longer survival in PDX mice than single therapy. These results shed light on the role of decitabine on tumor microenvironment re-modulation of methylation profile of promoter genes and suggest that PD-1 blockade and low-dose decitabine would be effective in future clinical trials.

Huang also correlated decitabine and anti-PD-L1 treatment to colorectal cancer (Huang et al., 2020). Using *in vitro* and *in vivo* models, the study showed that decitabine induces DNA hypomethylation which enhances tumor PD-L1 expression *via* an epigenetic mechanism, improving the therapeutic efficacy of anti-PD-L1 immunotherapy. Besides, decitabine treatment modifies the interferon signaling pathway and remodels the tumor microenvironment, recruiting more immune cells, such as T cells for antitumor immunity (Huang et al., 2020).

An important work performed by Goltz used data from 470 melanoma patients provided by The Cancer Genome Atlas and a cohort of 50 metastatic melanoma patients treated with anti–PD-1 or/and anti–CTLA-4 antibodies, to investigate if *CTLA-4* promoter methylation profile can be used as a biomarker to predict more successful treatment with ICB (immune checkpoint blockage). Methylation levels in patients treated with anti-PD-1 and anti-CTLA-4 were slightly lower when compared to the non-ICB cohort. Low *CTLA-4* methylation levels also play a key role in prolonged overall survival observed in patients (Goltz et al., 2018). The study paves the way for use of demethylating agents to aid the treatment with immune checkpoint inhibitors. Several clinical trials have been performed to evaluate the effects of the combination of epigenetic inhibitors and immunotherapies which creates hope for more efficient treatments and overcomes the limitations of current approaches (Topper et al., 2020; Villanueva et al., 2020; Licht and Bennett, 2021).

### Epi-cell therapy

Chimeric Antigen Receptor (CAR-T) cell therapy has revolutionized personalized cancer treatment. Some of the strategies available for this approach target B cell tumors, through molecular markers such as CD19, CD20, and CD22. DNA methylation profiles have been reported to impact outcomes of CAR-T targeting CD19 treatment. A study revealed that the use of the DNA methylation inhibitor 5-Aza-2′-deoxycytidine in the hypermethylated T-cell lines resulted in the downregulation of *INPP5A* and *ECHDC1* expression levels. These genes are involved in intracellular signaling cascades, important to CAR-T Cell therapy. In addition, these T-cell–derived lines also showed that hypermethylation of 5′-end CpG sites was associated with transcript downregulation. An illustrative example is the 5′-UTR CpG hypermethylation of *FOXN3*, a candidate tumor suppressor gene for T-cell acute lymphocytic leukemia which was downregulated in the T-cell-derived lines mentioned (Garcia-Prieto et al., 2022). Wang discussed the influence of decitabine, a DNMTi, on T-cell exhaustion of CAR-T therapy. Decitabine-treated CAR-T cells (dCAR) differentially expressed genes regarding proliferation, cytokine secretion, cytotoxicity and memory *in vitro*. dCAR also presented tumor shrinkage in the acute lymphoblastic leukemia mouse model (Wang et al., 2021). Xu reveal the benefits of priming CAR-T cell therapy mice with 5-azacytidine, a DNMTi. CD19^+^ B-cell acute lymphoblastic leukemia mouse models were used to perform those experiments. A regimen of 1 day of azacitidine before CAR-T cell infusion expanded IFNγ^+^ effector T cells and promoted CAR-T cell divisions. Azacitidine was related to activating several immune pathways, such as *TNFSF4*, a gene that encodes OX40L which is related to co-stimulatory signals on CAR-T cells. Another interesting finding is that neither PD-L1/PD-L2 expression in leukemia cells nor PD-1 expression in CAR-T cells was affected by pre-treatment with azacitidine, suggesting that azacitidine effects were not mediated through the modulation of the inhibitory PD-1 checkpoint (Xu et al., 2021). Thus, the study of the epigenetics landscape in CART-T-Cells can improve the efficacy of the cellular immunotherapy treatment in patients with B-cell malignancy and expanding its use to other oncological diseases.

## Conclusion

Epigenetic reprogramming is the main event to promote cell differentiation, and once cell fate is determined, the epigenetic pattern of genomic function must be stably maintained during DNA replication in cell division. Disruption of the epigenetic landscape of differentiated cells changes cell fate and promotes carcinogenesis and tumor progression. Thought waves of reprogramming cancer cells have the ability to return to a less differentiated state or aberrantly reprogram the stem progenitor. This stem cell-like phenotype is a challenge in cancer treatment leading to resistance, recurrence, and poor overall survival. Loss of global DNA methylation, aberrant activation/inactivation of growth-related genes, altered chromatin remodeling through histone modifications and ncRNA interaction, as well as disrupted expression of microRNAs and lncRNA, are molecular features of many cancer types. Given the heterogeneity of tumor cells, epigenetic changes have been highlighted as an important diagnostic marker, even in the early stages of cancer development, with great prognostic value. Recently, ncRNAs have an emerging role as a less invasive biomarker for diagnosis since they can be easily detected in body fluid and liquid biopsies. The complex regulatory machinery involved in the establishment and maintenance of epigenetic markers, and the cell-type specific modifications, give an individual variation in oncology, highlighting the importance of precision medicine. In this field, regulating the enzymes that catalyze epigenetic modifications using inhibitors or compounds that target these modifications has been extensively used in cancer therapy, especially for hematological tumors, although some of them are used for solid tumors as combinatory therapy. DNMTi and HDACi are the main epidrugs in clinical use and other new classes of epigenetic modulators are in development. Even though it has great results as monotherapy, the synergic use with other anticancer therapies, such as chemo, hormonal or immunotherapy has expanded the potential and effectiveness of epidrugs on cancer treatment.
